# A simplified mathematical model of directional DNA site-specific recombination by serine integrases

**DOI:** 10.1098/rsif.2016.0618

**Published:** 2017-01

**Authors:** Alexandra Pokhilko, Jia Zhao, W. Marshall Stark, Sean D. Colloms, Oliver Ebenhöh

**Affiliations:** 1 Institute of Molecular, Cell and Systems Biology, University of Glasgow, Glasgow G12 8QQ, UK; 2 Cluster of Excellence on Plant Sciences (CEPLAS), Heinrich-Heine-University, Universitätsstraße 1, 40225 Düsseldorf, Germany

**Keywords:** site-specific recombination, mathematical model, serine integrase, directionality

## Abstract

Serine integrases catalyse site-specific recombination to integrate and excise bacteriophage genomes into and out of their host's genome. These enzymes exhibit remarkable directionality; in the presence of the integrase alone, recombination between *attP* and *attB* DNA sites is efficient and irreversible, giving *attL* and *attR* products which do not recombine further. However, in the presence of the bacteriophage-encoded recombination directionality factor (RDF), integrase efficiently promotes recombination between *attL* and *attR* to re-form *attP* and *attB*. The DNA substrates and products of both reactions are approximately isoenergetic, and no cofactors (such as adenosine triphosphate) are required for recombination. The thermodynamic driving force for directionality of these reactions is thus enigmatic. Here, we present a minimal mathematical model which can explain the directionality and regulation of both ‘forward’ and ‘reverse’ reactions. In this model, the substrates of the ‘forbidden’ reactions (between *attL* and *attR* in the absence of RDF, *attP* and *attB* in the presence of RDF) are trapped as inactive protein–DNA complexes, ensuring that these ‘forbidden’ reactions are extremely slow. The model is in good agreement with the observed *in vitro* kinetics of recombination by ϕC31 integrase, and defines core features of the system necessary and sufficient for directionality.

## 1. Introduction

**Figure 1.**
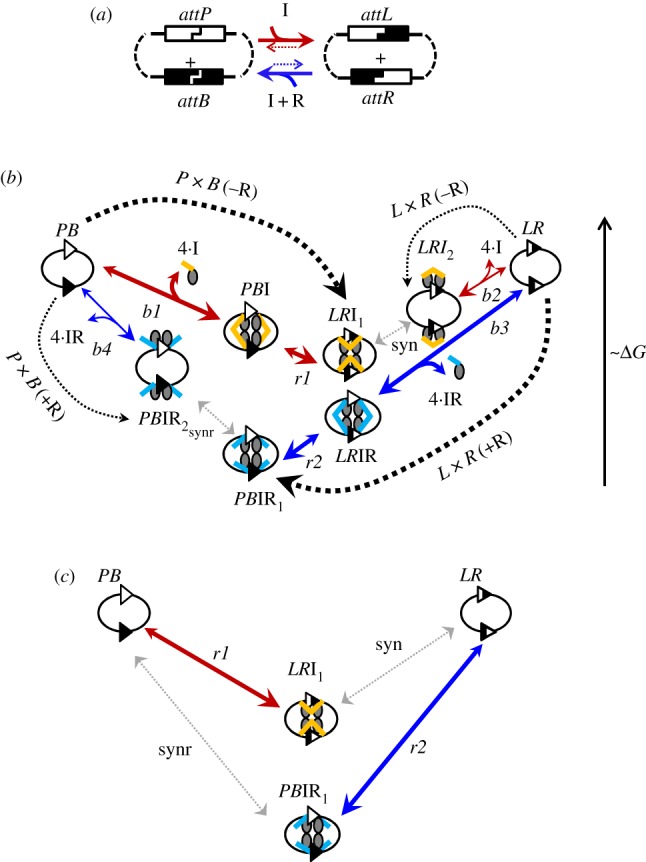
Schematic representations of serine integrase-mediated recombination reactions. (*a*) Overview, showing integrase (I) reacting to convert a *PB* (*attP* + *attB*) substrate to *LR* (*attL* + *attR*) product in the absence of RDF (red), whereas integrase plus RDF (I + R) converts *LR* (*attL* + *attR*) to *PB* (blue). (*b*) Scheme of reaction steps in Model M. Blue and red solid arrows show integrase-catalysed steps with and without RDF respectively. *PB*I, *PB*IR_1_, *PB*IR_2_ and *LR*I_1_, *LR*I_2_, *LR*IR are complexes containing four molecules of the integrase protein with *PB* or *LR* DNA substrates (with or without four molecules of RDF). The *P* × *B*(−R) reaction starts with the binding of four molecules of integrase (I) to *PB* substrate, followed by a recombination step and formation of the final product *LR*I_1_. The *L* × *R*(+R) reaction starts with the binding of four molecules of an integrase–RDF complex (IR) to *LR* substrates, followed by a recombination step and formation of the final product *PB*IR_1_. The ‘forbidden’ *P* × *B*(+R) and *L* × *R*(−R) reactions form ‘blocked’ *LR*I_2_ and *PB*IR_2_ complexes, which delay recombination due to their very slow conformational change to the productive *LR*I_1_ and *PB*IR_1_ complexes (grey dotted arrows). The favourable directions of reaction steps are shown by big arrowheads. Step names are shown near arrows. The cartoons show hypothetical structures of intermediates; note that alternative structures might be involved. See [1] for further details; coiled-coil domains of integrase shown by yellow sticks (or by blue sticks in presence of RDF). (*c*) Core structure of reactions after reduction of the fast variables. Substrates and products are shown by cartoons. Very slow steps are indicated by grey lines.

Serine integrases catalyse integration of a circular bacteriophage genomic DNA molecule into the bacterial host chromosomal DNA, by recombination between an *attP* site in the phage DNA and an *attB* site in the host DNA. In the resulting ‘lysogenic’ state, the phage genome is integrated in the host genome, and is flanked by recombinant *attL* and *attR* sites, each consisting of an *attP* half and an *attB* half (figure 1*a*). To resume its replicative life cycle, the phage DNA must be excised from its bacterial host genome. To accomplish this, a phage-encoded recombination directionality factor (the RDF protein) is expressed together with the integrase protein. RDF interacts with integrase and alters its properties so that it recombines the *attL* and *attR* sites to release the circular phage genomic DNA with an *attP* site, and leave an *attB* site in the host genome (figure 1*a*). (Hereafter, we refer to recombination between *attP* and *attB* as *P* × *B* recombination, and recombination between *attL* and *attR* as *L* × *R* recombination.)

Serine integrase-mediated recombination can be reconstituted *in vitro*, using purified integrase, RDF and DNA substrates [2–5]. The *in vitro* reactions strikingly reproduce the directionality observed *in vivo*. *P* × *B* recombination is efficient (i.e. most of the substrate molecules are recombined) when only integrase protein is present, and the reaction is unidirectional (i.e. no *L* × *R* recombination is observed under these conditions). However, when RDF is also present, *L* × *R* recombination is efficient and most of the sites are converted to *attP* and *attB* products. In addition to stimulating the *L* × *R* reaction, the presence of RDF inhibits the *P* × *B* reaction. No high-energy cofactors (such as adenosine triphosphate) are needed for recombination, and the unbound DNA substrate and product molecules are expected to be approximately isoenergetic. The molecular basis of the thermodynamic ‘driving force’ that favours *P* × *B* recombination in the absence of RDF, but *L* × *R* recombination in the presence of RDF, is unknown.

Serine integrases have recently attracted much attention as potential tools for experimental and applied genetic manipulations, because of their recombination efficiency, their short DNA recombination sites (*att* sites) (typically 40–50 bp) and absence of host factor requirements [5,6]. Mathematical and biochemical analysis of recombination directionality in these systems is therefore timely. We recently presented a detailed mathematical model of recombination by ϕC31 integrase (the first serine integrase to be identified, and the best-characterized to date) [5,7,8], which aimed to account as far as possible for the available biochemical, molecular and structural data [1] (here called ‘Model A’). This model comprises 35 ordinary differential equations (ODEs). Although it provides a good match to the *in vitro* kinetics data, its complexity makes it difficult to identify the key steps that determine directionality. Additionally, the large number of parameters in our previous model complicates analysis of their specific effects on reaction kinetics. We were therefore motivated to create a highly simplified data-driven mathematical model of serine integrase-mediated recombination. Such a minimal model with a simple structure and minimal number of parameters should be useful in analysis of the key principles of unidirectional reversible genetic transformations, which might be applicable to other biological systems.

Here, we present our simplified model, which consists of only three ODEs and assumes that all other steps of the reaction are in rapid quasi-equilibrium. This simplified model clearly illustrates the key theoretical assumptions required for the directionality and regulation of recombination by serine integrases. Moreover, owing to its simplicity, this model is more generally applicable and is easily adaptable to other integrase-mediated recombination systems.

## 2. Model description

**Figure 2.**
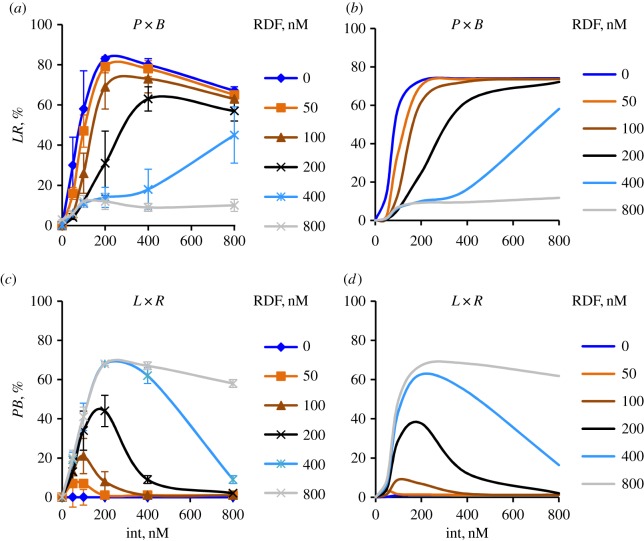
Dependence of ϕC31 integrase-mediated recombination on concentration of the RDF gp3. The product levels were calculated/measured after 3 h of *P* × *B* (*a*,*b*) and *L* × *R* (*c*,*d*) reactions with varying integrase and RDF concentrations, in Model M (*b*,*d*) and as observed experimentally (*a*,*c*). Experimental data are replotted from [1]. Total content of plasmid DNA substrate was 10 nM in all cases. Different lines correspond to different concentrations of RDF, as indicated.

The minimal model (referred to hereafter as ‘Model M’) was fitted to *in vitro* experimental data on recombination by ϕC31 integrase (I) with its RDF gp3 (R) [1] (figure 2). In the experiments used to produce these data, the extent of recombination at different concentrations of integrase and RDF was determined after 3 h reactions. Recombination was intramolecular, between *attP* and *attB* sites (*PB*) or *attL* and *attR* sites (*LR*) in inverted repeat orientation on supercoiled plasmid substrates. Recombination inverts the orientation of the DNA sequence flanked by the *att* sites, but the size of the plasmid is unchanged. Model M therefore assumes intramolecular recombination of a *PB* or *LR* plasmid substrate, but it would be easily adaptable for other types of substrates. The reaction scheme is a simplification of that used in the previously reported full model, Model A [1] (figure 1*b*). Model M consists of a set of reversible reaction steps, each described by first or second order kinetics.

The *P* × *B*(−R) reaction starts by binding of four molecules of I to the *PB* substrate (two molecules to each *att* site) and formation of the *PB* synapse (*PB*I), in which the *attP* and *attB* sites in the *PB* plasmid are held together by an integrase tetramer. Model M describes the formation of *PB*I synapse from free *PB* substrate as a single binding step (step ‘*b1’*), although the process requires multiple molecular events, which were represented as two steps (*b1* and *s1*) in our earlier Model A. This simplification was based on our earlier assumption that integrase binding is faster than synapsis and therefore is not a rate-limiting step [1]. The next step (recombination ‘*r1’*) transforms *PB*I to *LR*I_1_, comprising recombinant *attL* and *attR* sites bound together by an integrase tetramer which synapses the two sites (figure 1*b*). Here again, this single step describes a series of molecular events (*r1* and *mod* in Model A). Even though the rate of step *mod* in Model A is unknown, it was reasonable to assume that it is faster than the recombination step *r1*, which includes multiple complex changes in DNA state, such as cleavage, rotation and strand exchange [1,5,6]. Model M proposes that *LR*I_1_ is the predominant endpoint of the *P* × *B*(−R) reaction at short reaction times (of the order of 3 h), because the next step on the pathway is very slow (see below).

In Model M, the ‘forbidden’ *attL* × *attR* reaction in the absence of RDF (*L* × *R*(−R)) starts with the binding of four I molecules to *LR* (step *b2* in figure 1*b*), forming the *LR*I_2_ complex. Crucially, the *LR*I_2_ complex is conformationally distinct from *LR*I_1_, the immediate product of *P* × *B* recombination. Conversion of *LR*I_2_ into *LR*I_1_ (step ‘syn*’*) and its reverse are assumed to be very slow reactions, of the order of days (as in Model A where it is referred to as step *s2*). A hypothetical structure-based interpretation of these two complexes has been presented [1] (figure 1*c*). The very slow interconversion of *LR*I_1_ and *LR*I_2_ complexes is the key feature that explains why the *L* × *R*(−R) reaction does not yield detectable levels of *PB* recombination product in short reactions (of the order of hours).

In Model M, the reaction of the *LR* substrate in the presence of RDF (*L* × *R*(+R)) starts by binding of integrase–RDF complexes to the *attL* and *attR* sites. We assume (based on published data) that each integrase monomer interacts in solution with one RDF monomer to form a 1:1 complex (IR) [1,3,9]. Therefore, four IR complexes bind to *LR*, forming a synaptic complex *LR*IR (figure 1*b*, step ‘*b3’* (steps *b3* and *s3* in Model A)). Synapsis is followed by strand exchange step ‘*r2’* (steps *r2* and *modr* in Model A), forming a *PB* product synapse comprising IR-bound recombinant *attP* and *attB* sites, *PB*IR_1_ (figure 1*b*). Analogously to our hypothesis for *P* × *B*(−R) recombination (see above), we propose that *PB*IR_1_ is the typical endpoint of the *L* × *R*(+R) reaction. In the ‘forbidden’ *P* × *B*(+R) reaction, we assume that a different complex *PB*IR_2_ is formed by binding of four IR complexes to *PB*, and that conversion of *PB*IR_2_ to *PB*IR_1_ (step ‘synr*’*) and its reverse (equivalent to *s4* in Model A) are very slow.

Model M also includes the possible formation of unproductive complexes which contain a synaptic integrase tetramer, but fewer than four RDF molecules (*PB*IR*_i_*, *LR*IR*_i_*; not shown on figure 1*b*, for clarity). For simplicity, the model only includes one representative version of each species, one for LR and one for PB, each containing two RDF and four integrase monomers. These two complexes correspond to multiple unproductive complexes in the full model (Model A) and are assumed to be completely unproductive for recombination. The inclusion of complexes *PB*IR*_i_* and *LR*IR*_i_* in Model M was sufficient to describe the sharp response of the reactions to small changes in the ratio of RDF:integrase protein concentrations when this ratio is close to 1 (figure 2).

We assume that all reaction steps except recombination (*r1*, *r2*) and the slow synaptic conformational change steps (syn, synr) are fast. This assumption allows us to reduce the dimensionality of the model by applying rapid equilibrium approximations to the other steps, including formation of integrase–RDF complex IR in solution and binding of integrase (with or without RDF) to DNA. After these simplifications, four slowly changing variables (the concentrations of *LR*I_1_, *PB*IR_1_, total *PB* and total *LR)* remain, of which only three are independent, because of conservation of the total DNA pool. We chose the three quantities *LR*I_1_, *PB*IR_1_ and total *PB* as the independent slow dynamic variables, with total *LR* being determined by the difference between total DNA and total *PB*.

The concentrations of rapidly equilibrating species (*PB, PB*I, *PB*IR_2_, *LR*, *LR*I_2_, *LR*IR, as well as IR and the inhibitory complexes *LR*IR*_i_* and *PB*IR*_i_*) can be analytically determined from the slowly changing variables along with the conserved total concentrations of integrase (I_tot_), RDF (R_tot_) and DNA (D_tot_). Applying the rapid equilibrium approximation yields expressions for the concentrations of the equilibrating complexes as functions of free I, R, *LR* and *PB* as follows:
2.1
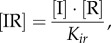

2.2
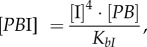

2.3
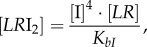

2.4
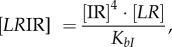

2.5
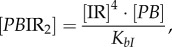

2.6
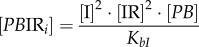

2.7
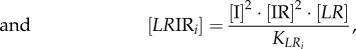
where *K_ir_, K_bI_* and 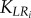 are the dissociation constants for the respective complexes.

For simplicity and because it was sufficient to describe the data (figure 2), we assume that all of the complexes of integrase (or integrase and RDF) with DNA (complexes formed in steps *b1, b2, b3, b4*, and also unproductive complex *PB*IR*_i_*) have the same dissociation constant *K_bI_*. The dissociation constant 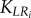 for the unproductive DNA complex *LR*IR*_i_* was required to be lower than *K_bI_*. This is a consequence of the simplified assumption that there is only one type of unproductive LR-integrase–RDF complex (*LR*IR*_i_*), with two molecules of RDF per four molecules of integrase (in Model A there were three unproductive complexes, with one, two and three molecules of RDF). A low value of 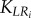 prevents formation of *PB* product in the *L* × *R*(+R) reaction when RDF concentrations are lower than integrase, in agreement with the data [1], which show sharply reduced *L* × *R*(+R) recombination when RDF is lower than integrase (figure 2).

The total concentrations of integrase and RDF can be expressed as the sum of the concentrations of all complexes containing these species
2.8′
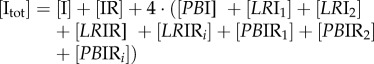


and
2.9′
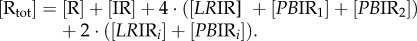


While correct expressions for [I] and [R] can in principle be derived, their analytic forms are highly complex. We therefore make the approximation that the concentrations of DNA-bound integrase and RDF species are negligible. Thus considering only unbound integrase and RDF
2.8
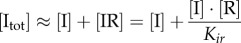
and
2.9
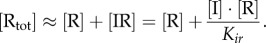


This approximation is justified by the experimental observations that the concentrations of integrase and RDF required for efficient recombination are typically much higher (more than 10-fold) than the concentration of the DNA substrate. For example, in our experimental conditions with DNA concentrations of 10 nM, integrase concentrations above 200 nM were required for efficient recombination [1]. Thus, even if every plasmid binds four integrase molecules (two molecules to each *att* site), the free integrase pool is reduced by only 20%.

From the above equations, R is easily expressed via I_tot_ and R_tot_
2.10
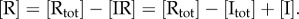
Inserting equation (2.10) into equation (2.8) leads to the quadratic equation

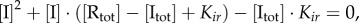
resulting in
2.11
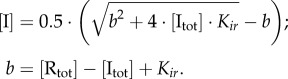
The total concentration of *PB* can be expressed as a sum of the concentrations of all species containing *PB*

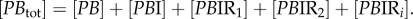


After expressing the concentrations of all quickly changing variables via [I], [R], [*PB*] (equations (2.1)–(2.7)), we can get the following expression for [*PB*]:
2.12
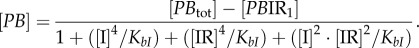
Similarly, the total concentration of DNA (D_tot_) can be expressed as the sum of concentrations of all PB and all LR containing species


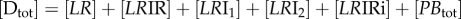
, leading to the equation
2.13
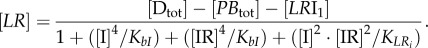
The kinetics of the slowly changing variables (the concentrations of *LR*I_1_, *PB*IR_1_, *PB*_tot_) are governed by the following three ODEs:
2.14
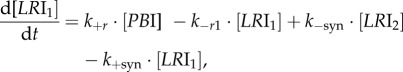

2.15
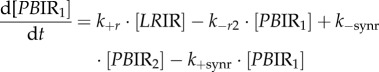



2.16
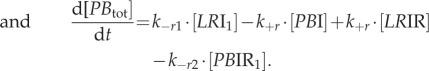


The parameters *k_+r_*, *k_+_*_syn_, *k_+_*_synr_ and *k_−r1_*, *k_−r2_*, *k_−_*_syn_, *k_−_*_synr_ are respectively the forward and reverse rate constants of the strand exchange steps (*r1*, *r2*) and modification steps (syn, synr), with the forward direction defined as *PB* → *LR* for (−R) and *LR* → *PB* for (+R) reactions. The forward rate constants of steps *r1* and *r2* are assumed to be equal (*k_+r_*), for simplicity. This assumption resulted in a good fit to our data (figure 2). Equilibrium constants *K_r1_*, *K_r2_*, *K*_syn_, *K*_synr_ are calculated as the ratios of forward and reverse rate constants: *K_eq_n_* = *k_+n_*/*k_−n_*. All concentrations are expressed in micromolars; the time units are hours.

Equations (2.1)–(2.16) comprise the complete set of equations describing the dynamics of the system. Concentrations were set to reflect the conditions used experimentally (figure 2). Thus, the total DNA concentration DNA_tot_ was 10 nM, while integrase and RDF concentrations were varied.

Model M includes 10 parameters (the seven forward and reverse rate constants from differential equations (2.14)–(2.16), the DNA binding dissociation constants *K_bI_*, 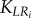 and the dissociation constant of IR, *K_ir_*) presented in electronic supplementary material, table S1. These parameters were chosen to fit the experimental data [1]. In particular, the equilibrium constants of the strand exchange steps *K_r1_*, *K_r2_* were fitted to the observed extent of recombination after 3 h of the *P* × *B*(−R) and *L* × *R*(+R) reactions under saturated integrase (and RDF for *L* × *R*(+R) reaction) concentrations. The rate constant of the slow step ‘synr’ (*k_−_*_synr_) was fitted to the observed extent of recombination after 3 h of the ‘forbidden’ *P* × *B*(+R) reaction (figure 2). For the rate constant of the ‘syn’ step (*k_−_*_syn_), only the upper bound was estimated based on the observed absence of recombination after 3 h of the ‘forbidden’ *L* × *R*(−R) reaction (figure 2). The rate constants of strand exchange steps were fitted to the observed rates of recombination (see the Results section). Finally, the equilibrium constants *K*_syn_ and *K*_synr_ were determined from the conservation of energy during the *P* × *B* and *L* × *R* reactions, which requires that the product of all equilibrium constants of the reactions equals 1 according to Wegscheider's condition [10]:

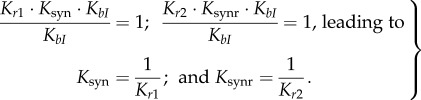


The presence of these constraints results in a balance between energetically favourable and non-favourable steps. For example, the favourable binding of integrase to *PB* substrate and formation of the *LR*I_1_ product of the *P* × *B*(−R) reaction is balanced by the unfavourable transition from *LR*I_1_ to *LR*I_2_ and dissociation of integrase from *LR*I_2_ (figure 1*b*).

The system of ODEs was solved using Matlab, integrated with the stiff solver ode15s (The MathWorks UK, Cambridge). Matlab code of the main model is provided in electronic supplementary material, text S1 and is freely available at https://github.com/ (the full URL is https://github.com/QTB-HHU/integraseModel/tree/master/minimalModel).

## 3. Results and discussion

**Figure 3.**
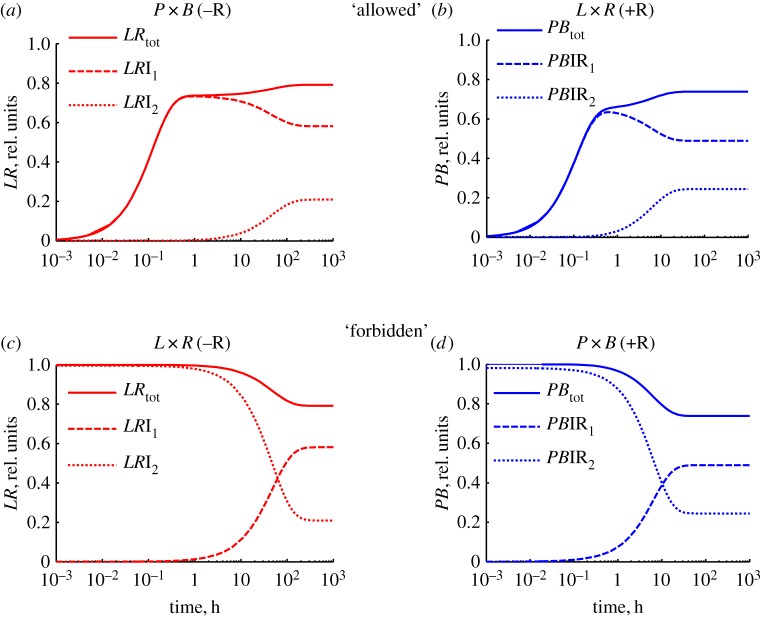
Simulated time courses of the amounts of abundant DNA-containing products. Panels (*a,b*) show the kinetics of the ‘allowed’ reactions (*P* × *B*(−R) and *L* × *R*(+R)), whereas panels (*c,d*) display the kinetics of the ‘forbidden’ reactions. Solid red and blue lines show the total amount of *LR* and *PB* plasmid molecules, respectively. The dotted and dashed lines show the amounts of integrase–DNA complexes as indicated at the left in each panel. The amounts of all DNA-containing species are expressed relative to the total DNA content. The calculations were performed for 10 nM of plasmid substrate, 400 nM of integrase and 800 nM of RDF (for panels (*b*) and (*d*)).

Our Model M is based on the simplified reaction scheme of figure 1*b*. Several steps in this scheme are formed by combining a number of steps in Model A [1]. For example, the formation of the substrate synapse *PB*I from free *PB* and four integrase molecules is considered as a single step in Model M, combining intermediate steps (binding of integrase monomers and/or dimers to individual *att* sites, and synapsis). In addition, the presence of the relatively slow recombination and desynapsis steps (*r1*, *r2*, syn and synr) allows us to apply rapid equilibrium approximations to the other reactions, further reducing the dimensionality of the model (figure 1*c*). As a result Model M has only three ODEs for the independent slowly changing variables, describing concentrations of *LR*I_1_, *PB*IR_1_ and conversion of *PB* to *LR* by the recombination steps (equations (2.14)–(2.16)), with other faster changing variables being expressed as functions of these slow changing ones. The initial rates of the ‘allowed’ recombination reactions are determined by the rates of the recombination steps (figure 3*a*,*b*), while the final approach to equilibrium is determined by very slow changes in the conformations of the product complexes, which might be related to slow desynapsis as proposed in [1] (figure 1*b*).

Despite the much reduced complexity of Model M compared to Model A, the key feature of different, slowly interconverting, tetrameric integrase complexes formed from either *PB* or *LR* substrates is preserved. The complex *LR*I_1_ is formed after strand exchange during the *P* × *B*(−R) reaction, whereas the complex *LR*I_2_ is formed when integrase is added to the ‘naked’ *LR* plasmid (*L* × *R*(−R) reaction; figures 1*b* and 3*a*,*c*). Similarly, the complex *PB*IR_1_ is formed after strand exchange during the *L* × *R*(+R) reaction, whereas the complex *PB*IR_2_ is formed upon binding of integrase–RDF to the *PB* plasmid (*P* × *B*(+R) reaction; figures 1*b* and 3*b*,*d*). The interconversion of *LR*I_2_ and *LR*I_1_ is assumed to be very slow, as is interconversion of *PB*IR_2_ and *PB*IR_1_ (figure 3). These slow interconversions, which may be interpreted as indicating a high activation energy barrier, allowed us to describe directionality (as in Model A). Thus, the (‘allowed’) reactions *P* × *B*(−R) and *L* × *R*(+R) quickly approach quasi-equilibrium, with the accumulation of *LR*I_1_ and *PB*IR_1_ complexes respectively, which can equilibrate only very slowly with the *LR*I_2_ and *PB*IR_2_ complexes (figure 3*a*,*b*). The ‘forbidden’ reactions *L* × *R*(−R) and *P* × *B*(+R) are predicted to be far from equilibrium for long times, due to initial accumulation of non-recombinant, catalytically inactive *LR*I_2_ and *PB*IR_2_ complexes respectively, which convert very slowly to the catalytically active *LR*I_1_ and *PB*IR_1_ complexes (figure 3*c*,*d*; note the logarithmic time scale). This trapping of the substrates of ‘forbidden’ reactions in the blocked *LR*I_2_ and *PB*IR_2_ complexes makes the reactions practically irreversible *in vivo*, because such complexes will usually be destroyed by cellular processes such as DNA replication before they can convert to *LR*I_1_ or *PB*IR_1_ and undergo recombination.

Model M provides a good match to the *in vitro* experimental data on the kinetics of recombination by ϕC31 integrase and its RDF gp3 [1] (figure 2). In these experiments, the levels of recombinant products were measured after 3 h of the *P* × *B* and *L* × *R* reactions under different concentrations of integrase and RDF (figure 2*a*,*c*). The simulations with Model M quantitatively describe the key features of the data, such as the observed sharp stimulation of the *L* × *R*(+R) reaction (figure 2*c*,*d*) and inhibition of the *P* × *B*(−R) (figure 2*a*,*b*) reaction when the concentration of RDF reaches that of integrase. These effects are predicted to result from competition between RDF-containing and RDF-free complexes of integrase for binding to DNA substrates. Thus, *LR* substrate forms the blocked *LR*I_2_ complex when integrase is present in the absence of RDF, but increasing RDF concentration shifts the balance towards the productive *LR*IR complex (figures 1 and 2*c*,*d*). Similarly, *PB* substrate forms the productive *PB*I complex in the absence of RDF, but the blocked (RDF-containing) *PB*IR_2_ complex becomes predominant as RDF concentration is increased (figure 2*a*,*b*).

Certain minor features of our experimental data, which were accounted for in the full model (Model A), are not described by Model M, due to its simplicity. These features include the observed slight decrease of the maximal amount of recombination products when integrase concentrations are raised to higher than 200 nM (figure 2). This was described in Model A by the inclusion of unproductive complexes of integrase tetramers bound at single recombination sites. Also, Model M does not describe the observed small deviation of the initial kinetics of the reactions from simple exponential kinetics (electronic supplementary material, figure S1*a*). The observed two-exponential kinetics is better described by Model A (electronic supplementary material, figure S1*b*) because two steps (synapsis and recombination) limit the faster and slower exponentials respectively.

**Figure 4.**
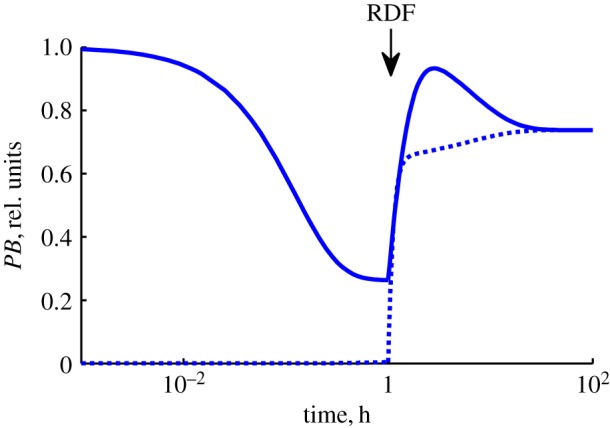
Simulated effect of addition of RDF (R) to the *P* × *B*(−R) reaction after 1 h (solid line). The kinetics of the *L* × *R*(+R) reaction is shown for comparison by a dotted line. The computations were performed with 10 nM plasmid DNA, 400 nM integrase and 800 nM RDF. (Online version in colour.)

As described above, our model predicts that two distinct *LR* products (*LR*I_1_ and *LR*I_2_) are formed depending on whether integrase is added to *PB* or *LR* DNA. However, these different products have not yet been detected experimentally. Model M makes a prediction that could allow us to test experimentally our hypothesis for the directionality of recombination. According to the model, when integrase is added to *PB* for 1 h, most of the DNA will form the *LR*I_1_ product. The addition of RDF at this point leads to reversal of the recombination step and rapid equilibration with the energetically more favourable *PB*IR_2_ complex. This leads to accumulation of *PB* DNA to levels that transiently approach 100% (figure 4; solid line), by the reverse of the *P* × *B*(−R) pathway. By contrast, if integrase is added to *LR* to form *LR*I_2_, addition of RDF after 1 h leads to formation of *PB*IR_1_ via the *L* × *R*(+R) pathway. This reaction never produces more than 74% *PB* product (figure 4; dotted line). Such high levels of *PB* product in a RDF-mediated reaction (near 100%) have not been observed to date in reactions catalysed by ϕC31 integrase, and would provide new evidence for our model for directionality.

**Figure 5.**
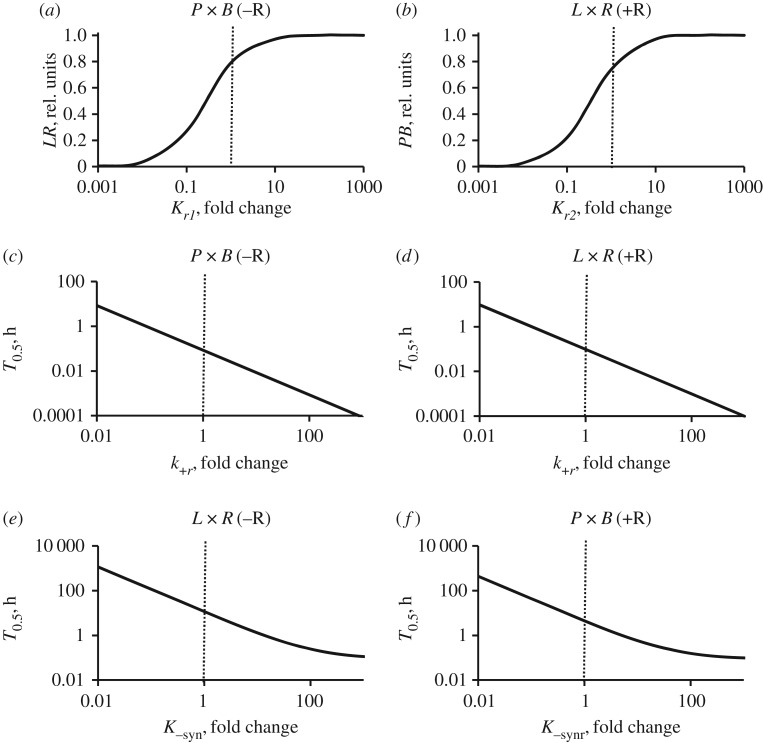
Effects of changes in Model M parameters on the key characteristics of integrase reactions. Simulations with unchanged parameters are indicated by dotted lines. (*a*,*b*) Dependence of extent of recombination at equilibrium on the equilibrium constants of ‘recombination’ steps, *K_r1_* (*P* × *B*(−R) reaction, (*a*)) or *K_r2_* (*L* × *R*(+R) reaction, (*b*)). (*c*–*f*) Dependence of ‘half-time’ (*T*_0.5_) (the time required to reach 50% of the maximum attainable product level) on the rate constant of the ‘recombination’ steps (*k_+r_*) for permitted reactions (*P* × *B*(−R), (*c*) and *L* × *R*(+R), (*d*)) or on the rate constants of the conformational changes (*k_−_*_syn_, *k_−_*_synr_) for non-permitted reactions (*L* × *R*(−R), (*e*) and *P* × *B*(+R), (*f*)). Changes in *K_r1_* or *K_r2_* were accompanied by compensating changes in the equilibrium constants of slow steps (*K*_syn_ or *K*_synr_ respectively), to maintain agreement with the energy conservation equations. Changes in the rate constants (*k_+r_*, *k_−_*_syn_ and *k_−_*_synr_) were accompanied by equal changes in the reverse rate constants (*k_−r1_*, *k_−r2_*, *k*_syn_ and *k*_synr_) to keep the equilibrium constants unchanged for these steps. The computations were performed for 10 nM substrate, 400 nM integrase and 800 nM RDF (for *b*,*d*,*f*).

We next used our model to explore the effects of different reaction steps on the overall kinetics. For practical applications, the recombination efficiency (that is, the maximum extent of conversion of substrate to product) is especially important; this can vary dramatically between different integrases [11]. Our analysis demonstrates that the reactions are most critically affected by the equilibrium constants of the *r1* and *r2* steps (*K_r1_* and *K_r2_*), which combine various steps including DNA strand exchange and the subsequent modifications. Variation of *K_r1_* or *K_r2_* results in large changes in recombination efficiencies (figure 5*a*,*b*), suggesting that variations in the efficiencies of different integrases might be primarily due to differences in these steps. Another important characteristic of an integrase system is its reaction rate. Our model predicts that the rates of the ‘allowed’ *P* × *B*(−R) and *L* × *R*(+R) reactions should be critically dependent on the rates of the recombination steps (rate constant *k_+r_*; figure 5*c*,*d*). The rates of ‘forbidden’ reactions *L* × *R*(−R) and *P* × *B*(+R) are strongly dependent on the rate constants of the conformational change *k_−_*_syn_ and *k_−_*_synr_ (figure 5*e*,*f*).

## 4. Conclusion

The minimal model (Model M) for integrase-mediated DNA recombination that we have presented above is able to account for the puzzling directionality of these systems. Model M is much simpler than our previously reported model (Model A), with only three independent variables governed by ODEs, but it is sufficient to quantitatively describe the *in vitro* experimental data on the kinetics of recombination by ϕC31 integrase and its RDF gp3. The model explains the observed directionality by the formation of stable synaptic complexes as end products of the ‘allowed’ recombination reactions (with or without RDF; *LR*I_1_ and *PB*IR_1_; figure 1), and kinetically stable inactive complexes with the substrates of ‘forbidden’ reactions (*LR*I_2_ and *PB*IR_2_; figure 1). This might represent a mechanism by which phages avoid spontaneous ‘reversal’ of integration and excision reactions.

Our minimal model emphasizes the key features of integrase-mediated reactions that bring about directionality, and might serve as a paradigm for those studying other biological systems with directional properties.

## Supplementary Material

Supplementary figure S1, Table S1 and Matlab code of the model
