# Supplementary material for: A simplified mathematical model of directional DNA site-specific recombination by serine integrases
Source: J R Soc Interface. 2017 Jan;14(126):20160618. doi: 10.1098/rsif.2016.0618 (PMC5310728; doi:10.1098/rsif.2016.0618)
Supplement: Supplementary figure S1, Table S1 and Matlab code of the model [file rsif20160618supp1.pdf]

Supplementary Materials

A simplified mathematical model of directional DNA site-specific recombination by serine integrases

Alexandra Pokhilko, Jia Zhao, W. Marshall Stark, Sean D. Colloms, and Oliver Ebenhöh

Supplementary Figures

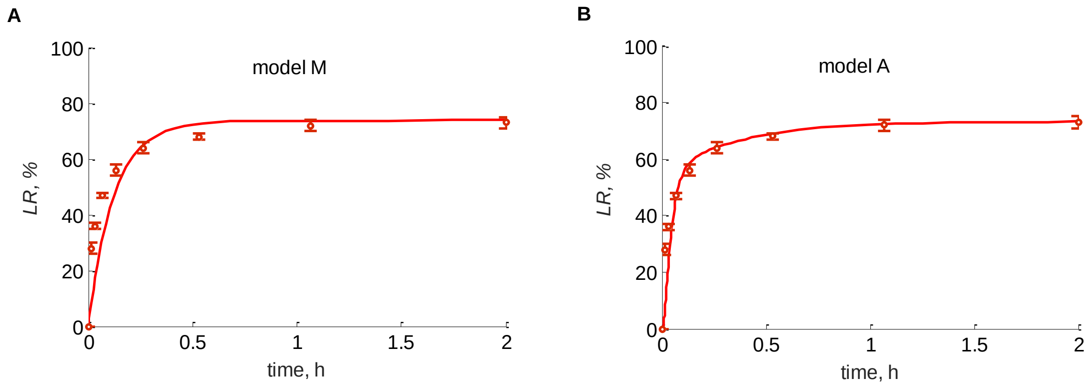

Figure S1. Comparison of the kinetics of the *P* × *B*(-R) reaction in minimal (Model M) (A) and full (Model A) (B) models. Reactions were with 10 nM of *PB* plasmid substrate and 400 nM integrase. The modelled data are shown by solid lines and experimental data by symbols (data taken from (1)). Error bars show standard deviations.

Supplementary Tables

Table S1. Parameter values of the model. The Table includes the forward rate constants k_{+r}, k_{+syn}, k_{+synr} (*PB*→*LR* direction for "-R" and *LR*→*PB* for "+R" reactions); the reverse rate constants k_{-r1}, k_{-r2}, k_{-syn}, k_{-synr}; the dissociation constant for the formation of *PB*I, *LR*I_2, *LR*IR, *PB*IR_2 and *PB*IRi complexes, K_{bI}; the dissociation constant of the formation of unproductive integrase-*LR* complex *LR*IRi, K_{LRi}; the dissociation constant of the formation of integrase-RDF complex IR in solution, K_{ir}. The dimensionless equilibrium constants of recombination and modification steps "*r1*", "*r2*", "*syn*", "*synr*", which are determined as $K_{eq\_n} = k_{+n} / k_{-n}$ for each step "n", are shown in brackets.

parameter	k_{+r}	k_{-r1}	k_{-r2}	k_{+syn}	k_{-syn}
value	6 h^{-1}	2.14 h^{-1} (K_{r1}=2.8)	3 h^{-1} (K_{r2}=2)	0.006 h^{-1}	0.017 h^{-1} (K_{syn}=0.35)
parameter	k_{+synr}	k_{-synr}	K_{bI}	K_{LRi}	K_{ir}
value	0.06 h^{-1}	0.12 h^{-1} (K_{synr} =0.5)	0.0001 μM^4	0.00002 μM^4	0.05 μM

Matlab code of the model

model file min_mod_1116.m

```
% main model file, calling for ode file Model_min_mod_1116.m

Kr1=2.8; Kr2=2; Kir=0.05; Ksyn=0.36; Ksynr=0.5; KbI=0.0001; Klri=0.00002;

% time unit - hour

% y(1)  BPtot
% y(2)  LR-int4 first
% y(3)  BP-int4-rdf2 first

Dtot=0.01; y0=[Dtot 0 0]; % initial conditions for PxB reaction
int_tot=0.4; rdf_tot=0.; %concentrations of integrase and RDF in mkM
%y0=[0 0 0]; rdf_tot=0.4; % initial conditions for LxR reaction

options = odeset();
   %options = odeset('MaxStep',0.0001);
b=rdf_tot-int_tot+Kir;
int=0.5*(sqrt(b*b+4*int_tot*Kir)-b);
rdf=rdf_tot-int_tot+int;

   t=[0 3]; % time interval
   [T, Y] = ode15s(@Model_min_mod_1116,t,y0,options,int,rdf,Dtot,Kir,Kr1,Kr2,Ksyn,Ksynr,KbI,Klri);

LRt=Dtot-Y(:,1);
Intrdf=int*rdf/Kir;
BP=(Y(:,1)-Y(:,3))/(1+int^4/KbI+Intrdf^4/KbI+int^2*Intrdf^2/KbI);
LR=(Dtot-Y(:,1)-Y(:,2))/(1+int^4/KbI+Intrdf^4/KbI+int^2*Intrdf^2/Klri);
BPI=BP*int^4/KbI;
```

```
LRI2=LR*int^4/KbI;
LRIR=LR*Intrdf^4/KbI;
BPIR2=BP*Intrdf^4/KbI;

% kinetics of the total LR and PB during PxB reaction; t=[0 3]; integrase=0.4 mkM, rdf=0
% to run LxR reaction, change initial condition to y0=[0 0 0]
% to calculate the product level at 3h, use the last datapoint of the product vector: LRt for PxB reaction; Y(:1) for LxR reaction
% to calculate reaction kinetics with other integrase or RDF concentration, change values of variables int_tot=0.4 and rdf_tot

figure (1)
plot(T,LRt/Dtot,'r');
hold on;
plot(T,Y(:,1)/Dtot,'b');
hold on;
title('LR_t-red; BP_t-blue');
```

the program uses the following function Model_min_mod_1116.m:

```
function Func = Model_min_mod_1116(t,y,int,rdf,Dtot,Kir,Kr1,Kr2,Ksyn,Ksynr,KbI,Klri);
% solving ODEs
Func = zeros(3, 1);

% y(1)  BPtot
% y(2)  LR-int4 first
% y(3)  BP-int4-rdf2 first

kpr=6;
kmr1=kpr/Kr1;
kmr2=kpr/Kr2;
kpsyn=0.006;
kmsyn=kpsyn/Ksyn;
kpsynr=0.06;
kmsynr=kpsynr/Ksynr;

intrdf=int*rdf/Kir;
Bp=(y(1)-y(3))/(1+int^4/KbI+intrdf^4/KbI+int^2*intrdf^2/KbI);
Lr=(Dtot-y(1)-y(2))/(1+int^4/KbI+intrdf^4/KbI+int^2*intrdf^2/Klri);
BpI=Bp*int^4/KbI;
LrI2=Lr*int^4/KbI;
LrIR=Lr*intrdf^4/KbI;
BpIR2=Bp*intrdf^4/KbI;

Func(1) = kmr1*y(2)-kpr*BpI+kpr*LrIR-kmr2*y(3);
Func(2) = kpr*BpI-kmr1*y(2)-kpsyn*y(2)+kmsyn*LrI2;
Func(3) = kpr*LrIR-kmr2*y(3)-kpsynr*y(3)+kmsynr*BpIR2;
```

To reproduce Figures, run the following scripts:

For Fig. 2B run file makeFig2B.m:

```
% run this file to reproduce Fig.2B
% Dependence of the level of LR product (% from max) after 3h of PxB reaction on concentration of integrase
% Different lines correspond to different concentrations of RDF: 0 (blue),
% 50 nM (red), 100 nM (yellow), 200 nM (black), 400 nM (magenda), 800 nM (green)

Dtot=0.01;
```

```
y0=[Dtot 0 0]; % initial conditions for PxB reaction

RDF = [0,0.05,0.1,0.2,0.4,0.8];
col = ['b','r','y','k','m','g'];

figure()
for r=1:length(RDF)

    rdf_tot=RDF(r); %concentrations of RDF
    %y0=[0 0 0]; rdf_tot=0.4; % initial conditions for LxR reaction
    for i=1:17
        x_int(i)=0.05*(i-1);
        int_tot=x_int(i);
        [Y,T]=min_mod_251116(rdf_tot, int_tot, y0, 3);
        PB=Y(:,1);
        LRt=Dtot-PB;
        LR_3h(i)=LRt(end)/Dtot*100;
    end

    plot(x_int*1000,LR_3h,col(r),'DisplayName',num2str(rdf_tot*1000));
    hold on;

end

xlabel('integrase, nM');
ylabel('LR, %');
title('LR product after 3h for different integrase and RDF concentrations (nM)')
legend('show')
```

For Fig. 2D run file makeFig2D.m:

```
% run this file to reproduce Fig.2D
% Dependence of the level of PB product (% from max) after 3h of LxR reaction on concentration of integrase
% Different lines correspond to different concentrations of RDF: 0 (blue),
% 50 nM (red), 100 nM (yellow), 200 nM (black), 400 nM (magenda), 800 nM (green)

Dtot=0.01;

y0=[0 0 0]; % initial conditions for LxR reaction

RDF = [0,0.05,0.1,0.2,0.4,0.8];
col = ['b','r','y','k','m','g'];

figure()
hold on;
for r=1:length(RDF)


    rdf_tot=RDF(r); %concentrations of RDF
    for i=1:17
        x_int(i)=0.05*(i-1);
        int_tot=x_int(i);
        [Y,T]=min_mod_251116(rdf_tot, int_tot, y0, 3);
        PB=Y(:,1);
        PB_3h(i)=PB(end)/Dtot*100;
    end
```

```
    plot(x_int*1000,PB_3h,col(r),'DisplayName',num2str(rdf_tot*1000));

end

xlabel('integrase, nM');
ylabel('PB, %');
title('PB product after 3h for different integrase and RDF concentrations (nM)')
legend('show')
```

For Fig. 3 run file makeFig3.m:

```
% run this file to reproduce Fig.3
% time courses of the most abundant DNA-containing products
% Panels A and B show the kinetics of the “allowed” reactions (PxB(-R) and LxR(+R))
% Panels C and D display the kinetics of the “forbidden” reactions (LxR(-R) and PxB(+R))

Dtot=0.01; Kr1=2.8; Kr2=2; Kir=0.05; Ksyn=0.36; Ksynr=0.5; KbI=0.0001; Klri=0.00002; int_tot=0.4;

figure()
% panel A
subplot(2,2,1)

y0=[Dtot 0 0]; % initial conditions for PxB reaction
rdf_tot=0; %concentrations of RDF
b=rdf_tot-int_tot+Kir;
int=0.5*(sqrt(b*b+4*int_tot*Kir)-b);
rdf=rdf_tot-int_tot+int;
Intrdf=int*rdf/Kir;
[Y,T]=min_mod_251116(rdf_tot, int_tot, y0, 1000);
PB=Y(:,1);
LRt=(Dtot-PB);
LR=(Dtot-Y(:,1)-Y(:,2))/(1+int^4/KbI+Intrdf^4/KbI+int^2*Intrdf^2/Klri);
LRI2=LR*int^4/KbI;

semilogx(T,LRt/Dtot,'r');
hold on;
semilogx(T,Y(:,2)/Dtot,'r--');
semilogx(T,LRI2/Dtot,'r:');
xlim([1e-3,1e3])
ylim([0,1])
ax=gca
ax.set('XTick',10.^[-3:3])
xlabel('time, h');
ylabel('LR, fraction from total DNA');
legend('LR_{tot}','LRI_1','LRI_2','Location','northwest')
title('"allowed" reaction PxB(-R)')

% panel B
y0=[0 0 0]; % initial conditions for LxR reaction
rdf_tot=0.8; %concentrations of RDF
b=rdf_tot-int_tot+Kir;
int=0.5*(sqrt(b*b+4*int_tot*Kir)-b);
rdf=rdf_tot-int_tot+int;
Intrdf=int*rdf/Kir;
[Y,T]=min_mod_251116(rdf_tot, int_tot, y0, 1000);
PBt=Y(:,1);
BP=(Y(:,1)-Y(:,3))/(1+int^4/KbI+Intrdf^4/KbI+int^2*Intrdf^2/KbI);
BPIR2=BP*Intrdf^4/KbI;
```

```
subplot(2,2,2)
semilogx(T,PBt/Dtot,'b');
hold on;
semilogx(T,Y(:,3)/Dtot,'b--');
semilogx(T,BPIR2/Dtot,'b:');
xlim([1e-3,1e3])
ylim([0,1])
ax=gca;
ax.set('XTick',10.^[-3:3])
xlabel('time, h');
ylabel('PB, fraction from total DNA');
legend('PB_{tot}','PBIR_1','PBIR_2','Location','northwest')
title('"allowed" reaction LxR(+R)')

% panel C
y0=[0 0 0]; % initial conditions for LxR reaction
rdf_tot=0.; %concentrations of RDF
b=rdf_tot-int_tot+Kir;
int=0.5*(sqrt(b*b+4*int_tot*Kir)-b);
rdf=rdf_tot-int_tot+int;
Intrdf=int*rdf/Kir;
[Y,T]=min_mod_251116(rdf_tot, int_tot, y0, 1000);
PB=Y(:,1);
LRt=(Dtot-PB);
LR=(Dtot-Y(:,1)-Y(:,2))/(1+int^4/Kbl+Intrdf^4/Kbl+int^2*Intrdf^2/Klri);
LRI2=LR*int^4/Kbl;

subplot(2,2,3)
semilogx(T,LRt/Dtot,'r');
hold on;
semilogx(T,Y(:,2)/Dtot,'r--');
semilogx(T,LRI2/Dtot,'r:');
xlim([1e-3,1e3])
ylim([0,1])
ax=gca;
ax.set('XTick',10.^[-3:3])
xlabel('time, h');
ylabel('LR, fraction from total DNA');
legend('LR_{tot}','LRI_1','LRI_2','Location','northwest')
title('"forbidden" reaction LxR(-R)')

% panel D
y0=[Dtot 0 0]; % initial conditions for PxB reaction
rdf_tot=0.8; %concentrations of RDF
b=rdf_tot-int_tot+Kir;
int=0.5*(sqrt(b*b+4*int_tot*Kir)-b);
rdf=rdf_tot-int_tot+int;
Intrdf=int*rdf/Kir;
[Y,T]=min_mod_251116(rdf_tot, int_tot, y0, 1000);
PBt=Y(:,1);
BP=(Y(:,1)-Y(:,3))/(1+int^4/Kbl+Intrdf^4/Kbl+int^2*Intrdf^2/Kbl);
BPIR2=BP*Intrdf^4/Kbl;

subplot(2,2,4)
semilogx(T,PBt/Dtot,'b');
hold on;
semilogx(T,Y(:,3)/Dtot,'b--');
semilogx(T,BPIR2/Dtot,'b:');
xlim([1e-3,1e3])
ylim([0,1])
```

```
ax=gca;
ax.set('XTick',10.^[-3:3])
xlabel('time, h');
ylabel('PB, fraction from total DNA');
legend('PB_{tot}','PBIR_1','PBIR_2','Location','northwest')
title('"forbidden" reaction PxB(+R)')
```

For Fig. 4 run file makeFig4.m:

```
% run this file to reproduce Fig.4
% effect of addition of RDF on PxB(-R) reaction. RDF added after 1 hour (solid line)
% The kinetics of the LxR(+R) reaction is shown for comparison by a dotted line
% The computations were performed with 400 nM integrase and 800 nM RDF

Dtot=0.01; Kr1=2.8; Kr2=2; Kir=0.05; Ksyn=0.36; Ksynr=0.5; KbI=0.0001; Klri=0.00002; int_tot=0.4;

y0=[Dtot 0 0];
rdf_tot=0;
[Y,T]=min_mod_251116(rdf_tot, int_tot, y0, 1);
PBt=Y(:,1);

figure()
p1=semilogx(T,PBt/Dtot,'b','DisplayName','PxB(-R) reaction');
hold on;

rdf_tot=0.8;
y0=[Y(end,1) Y(end,2) Y(end,3)];
[Y,T]=min_mod_251116(rdf_tot, int_tot, y0, 100);
PBt=Y(:,1);
T=T+1;

semilogx(T,PBt/Dtot,'b');

y0=[0 0 0];
rdf_tot=0;
[Y,T]=min_mod_251116(rdf_tot, int_tot, y0, 1);
PBt=Y(:,1);

p2=semilogx(T,PBt/Dtot,'b:','DisplayName','LxR(+R) reaction');

rdf_tot=0.8;
y0=[Y(end,1) Y(end,2) Y(end,3)];
[Y,T]=min_mod_251116(rdf_tot, int_tot, y0, 100);
PBt=Y(:,1);
T=T+1;

semilogx(T,PBt/Dtot,'b:');
xlabel('time, h');
ylabel('PB, fraction from total DNA');
legend([p1,p2],'PxB(-R) reaction','LxR(+R) reaction')
title('Simulated effect of addition of RDF after 1h')
```

The files, which reproduce the figures are calling for the funcion min_mod_251116.m:

```
function [Y,T] = min_mod_251116(rdf_tot, int_tot, y0,Tfin)
Dtot=0.01; Kr1=2.8; Kr2=2; Kir=0.05; Ksyn=0.36; Ksynr=0.5; KbI=0.0001; Klri=0.00002;

% time unit - hour
```

```
%y0 - initial conditions for the reaction
%int_tot, rdf_tot - concentrations of integrase and RDF in mkM

options = odeset();
   %options = odeset('MaxStep',0.0001);
b=rdf_tot-int_tot+Kir;
int=0.5*(sqrt(b*b+4*int_tot*Kir)-b);
rdf=rdf_tot-int_tot+int;

   t=[0 Tfin]; % time interval
   % kinetics of the reaction
      options = odeset('MaxStep',0.1);
   [T, Y] = ode15s(@Model_min_mod_1116,t,y0,options,int,rdf,Dtot,Kir,Kr1,Kr2,Ksyn,Ksynr,KbI,Klri);

PB=Y(:,1); % PB total
```

References

1. Pokhilko A, Zhao J, Ebenhoh O, Smith MC, Stark WM, Colloms SD. The mechanism of ϕC31 integrase directionality: experimental analysis and computational modelling. Nucleic Acids Res. 2016 Jul 7.
